# Supplementary material for: Research opportunities in precision oncology: perspectives on biospecimen and genomic data sharing from adults with cancer in Ireland
Source: Ir J Med Sci. 2025 Sep 26;194(6):1997–2008. doi: 10.1007/s11845-025-04082-4 (PMC12769628; doi:10.1007/s11845-025-04082-4)

## Supplementary data

| **Table S1. Spearman correlation analysis of educational attainment levels and respondent’s views on research & personal health.** Statistical significance is indicated as follows: *p < 0.05, **p < 0.01 |
| --- |


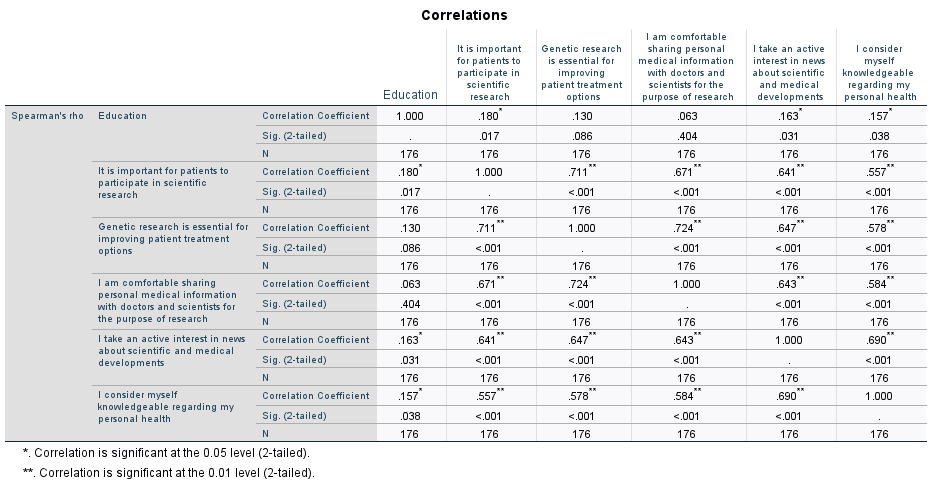

Supplement: Supplementary file 1 — Supplementary file1 (DOCX 64.7 KB) [file 11845_2025_4082_MOESM1_ESM.docx]
